# Assessment of localized brain regions correlated with MMSE using VBM analysis of structural MRI in a Japanese sample

**DOI:** 10.1016/j.ynirp.2025.100264

**Published:** 2025-04-29

**Authors:** Yoichi Sawada, Toru Satoh, Hideaki Saba, Yoshiki Kato, Tomoko Kuwada, Sayoko Shima, Kana Murakami, Megumi Sasaki, Yudai Abe, Kaori Harano

**Affiliations:** a Department of Contemporary Welfare, Faculty of Health and Welfare, Okayama Prefectural University, Japan; b Departments of Neurosurgery, Neurology, and Psychiatry, Ryofukai Satoh Neurosurgical Hospital, Japan; c Section of Higher Brain Function, Department of Rehabilitation, Ryofukai Satoh Neurosurgical Hospital, Japan; d Department of Human Welfare, Faculty of Human Relations, Otsuma Women's University, Japan

**Keywords:** Dementia, Gray matter volume, Mild cognitive impairment, Mini-mental state examination, Normal cognition, Structural magnetic resonance image, Voxel-based morphometry

## Abstract

The global aging population has led to a significant rise in dementia and cognitive decline, with Alzheimer's disease as the primary cause. Early detection of mild cognitive impairment (MCI), a prodromal stage of dementia, is critical for timely intervention. The Mini-Mental State Examination (MMSE) is commonly used for cognitive screening, yet its limitations—such as ceiling effects and educational biases—may hinder the early identification of subtle cognitive impairments. This cross-sectional study employed voxel-based morphometry (VBM) analysis of structural magnetic resonance imaging (MRI) to explore brain regions positively correlated with MMSE scores in a cohort of 510 participants. Significant gray matter volume (GMV) reductions were observed in the bilateral lateral frontal lobes, left medial frontal lobe, left hippocampus, left anterior cingulate cortex (ACC), and bilateral inferior temporal gyri in association with lower MMSE scores. Participants were classified into three groups—Normal Cognition (NC), MCI, and Dementia (D)—based on MMSE cutoff values. Compared to the NC group, the MCI group exhibited significant GMV reductions in the left hippocampus, left parahippocampal gyrus, left ACC, and right ventromedial prefrontal cortex (vmPFC). The D group showed further GMV reductions in the bilateral hippocampus and left inferior temporal gyrus compared to the MCI group. These findings highlight the clinical utility of VBM-based structural MRI in assessing localized brain atrophy associated with cognitive decline, supporting its potential role in early diagnosis and intervention for MCI. Further research integrating longitudinal studies and multimodal biomarkers is warranted to enhance diagnostic accuracy.

## 1 Introduction

The global incidence of dementia is rapidly increasing with the aging population, and it is projected that 150 million individuals will be affected by 2050 (GBD 2019 Dementia Forecasting Collaborators, 2022). Alzheimer's disease (AD), a progressive neurodegenerative disorder, is responsible for the majority of dementia cases, resulting in irreversible impairment of acquired cognitive functions. Mild cognitive impairment (MCI), characterized by cognitive decline, particularly memory deficits, represents a prodromal stage of dementia. Approximately 15–20 % of individuals with MCI progress to dementia each year (Petersen, 2009), though studies suggest that cognitive function may be restored in some MCI patients through appropriate treatment and intervention (Roberts and Knopman, 2013). Early intervention at the MCI stage—including exercise, cognitive training, and lifestyle modifications—has demonstrated efficacy in mitigating cognitive decline and may help preserve or enhance cognitive function (Ngandu et al., 2015). Furthermore, the recent clinical availability of disease-modifying therapies targeting amyloid plaques, such as Lecanemab and Donanemab (Volloch and Rits-Volloch, 2023), has underscored the urgent need for early detection of MCI and cognitive decline to facilitate timely therapeutic interventions. As a result, distinguishing MCI from Normal Cognition (NC) and Dementia (D) has become pivotal to the early diagnosis and management of cognitive impairments.

Voxel-based morphometry (VBM) analyses have consistently reported significant gray matter volume (GMV) reductions in the medial temporal lobe, including the hippocampus and entorhinal cortex, in MCI patients (Tabatabaei-Jafari et al., 2015). A systematic review with meta-analysis revealed that MCI patients exhibited a 2.2-fold higher volume loss in the hippocampus, 1.8-fold in the whole brain, and 1.5-fold in the entorhinal cortex compared to healthy controls (Tabatabaei-Jafari et al., 2015). Additionally, amnestic MCI patients display pronounced atrophy in the left amygdala and right hippocampus, regions associated with emotion, cognition, and memory (Zhang et al., 2021). In AD, GMV loss is more widespread and involves regions such as the entorhinal cortex, temporoparietal cortex, dorsolateral prefrontal cortex, occipital cortex, and precuneus (Derflinger et al., 2011; Minkova et al., 2017). Studies have suggested that GMV asymmetry in AD is more pronounced rather than lateralized, with a tendency for leftward atrophy in certain cortical areas (Derflinger et al., 2011). Furthermore, younger AD patients exhibit faster atrophy rates in the posterior brain regions, including the precuneus, parietal, and superior temporal lobes, which may be critical in understanding early-onset AD (Minkova et al., 2017). These findings indicate that GMV loss in MCI and AD follows a region-specific pattern, with early degeneration in the medial temporal lobe in MCI, followed by a more extensive cortical involvement in AD. Understanding these progressive atrophic changes is essential for identifying potential biomarkers and developing targeted interventions.

To date, neuropsychological tests that evaluate overall cognitive function have been used for screening cognitive impairments, with the Mini-Mental State Examination (MMSE) being one of the most widely used and straightforward tests worldwide (Mitchell, 2009). The cutoff score for dementia on the MMSE is set at 24/23 out of a total score of 30. However, MCI and NC often overlap within the 24–30 score range, raising concerns that the ceiling effect and educational bias of the MMSE may lead to the under-detection of subtle cognitive impairments or early MCI (Satoh et al., 2024; Sawada et al., 2023; Tsoy et al., 2021). Consequently, alternative approaches, such as low-cost and scalable digital cognitive assessments, are increasingly being explored as viable solutions to improve diagnostic accuracy in diverse populations (Tsoy et al., 2021). To enhance diagnostic accuracy, it is recommended to combine neuropsychological tests with biomarkers such as amyloid-beta and tau protein measured in cerebrospinal fluid and blood tests, as well as structural and functional imaging examinations, including MRI, SPECT, and PET (Dubois et al., 2023). Among these, structural MRI is non-invasive, safe, and cost-effective, providing high-resolution brain morphology information superior to that of functional imaging. Previous studies have demonstrated that regions such as the hippocampus, medial/lateral temporal lobe, and prefrontal cortex exhibit significant volume reduction in individuals with cognitive impairment, supporting their role as potential imaging biomarkers for MCI and AD (Risacher et al., 2010). Notably, in Japan, the widespread availability of MRI, supported by the universal health insurance system, ensures greater accessibility even in rural regions, setting it apart from many other countries (Matsumoto et al., 2015; Nature Research Custom Media, 2024). Given this infrastructure, MRI-based screening may be a more practical option in Japan compared to other healthcare systems. Although MRI is generally not feasible for large-scale cognitive screening worldwide, its accessibility in Japan offers a unique advantage for investigating the relationship between MMSE scores and brain structural changes in populations that have been relatively understudied.

Despite these advances, few studies have examined the relationship between neuropsychological test performance and brain volume in a Japanese population. Cultural, genetic, and environmental factors may influence these relationships, necessitating population-specific research. Integrating neuropsychological test scores with structural MRI findings can offer a more comprehensive understanding of the neurobiological basis of cognitive impairment. By leveraging Japan's high availability of MRI, this study aims to contribute to the validation of MMSE by assessing its association with structural brain changes using VBM analysis in a Japanese sample. The aims of this study were to statistically evaluate the localized brain regions with reduced GMV associated with declining MMSE scores across the entire sample, using VBM analysis of structural MRI, and to reveal distinct localized brain regions with gradual decline in GMV across the three groups stratified by MMSE scores (NC, MCI, and D groups). These analyses provide a detailed understanding of the relationship between cognitive function and brain structure, potentially informing improved clinical management strategies.

## 2 Materials and methods

This cross-sectional study was approved by the Ethics Committee of the Ryofukai Medical Corporation (Approval Number: IRB: 2019–01, Approval Date: January 16, 2019). All research procedures were conducted in accordance with the relevant ethical guidelines and regulations, and written informed consent was obtained from all participants.

### 2.1 Participants

The study included 510 participants (mean age ± standard deviation: 78.17 ± 9.09 years; 286 women and 224 men; mean educational level ± standard deviation: 1.94 ± 0.84) who presented with chief complaints such as memory loss and headaches and underwent neuropsychological screening for cognitive impairment, as well as structural MRI over a period of approximately four years (April 2019 to April 2023). Participants were stratified into three groups based solely on their MMSE scores: 199 individuals in the NC group (MMSE ≥28; mean age ± standard deviation: 74.59 ± 9.73 years; 105 women and 94 men; mean educational level ± standard deviation: 2.20 ± 0.86), 189 in the MCI group (MMSE 24–27; mean age ± standard deviation: 79.33 ± 7.43 years; 105 women and 84 men; mean educational level ± standard deviation: 1.85 ± 0.80), and 122 in the D group (MMSE ≤23; mean age ± standard deviation: 82.22 ± 8.17 years; 76 women and 46 men; mean educational level ± standard deviation: 1.66 ± 0.77). However, it is important to note that no clinical diagnosis by a neurologist was conducted in this study, which remains a limitation. Participants with visual, auditory, or motor impairments, as well as those with severe comorbid conditions that could interfere with test results, were excluded from the study. Sex was defined according to sex assigned at birth.

### 2.2 Neuropsychological assessment: MMSE

Cognitive impairment was screened using the Japanese version of the MMSE (MMSE-J) (Sugishita et al., 2018) for all participants. In line with the study objectives, subjects were classified into three groups based on MMSE cutoff scores: the NC group (MMSE ≥28), the MCI group (MMSE 24–27), and the D group (MMSE ≤23), as previously outlined.

### 2.3 MRI acquisition and preprocessing

Structural MRI of the head was conducted using a 3.0T MRI scanner (Signa Pioneer; GE Healthcare, Wisconsin, USA). The 3D-T1-weighted images (3D-T1WI) were acquired using the BRAVO sequence with the following parameters: TR/TE = 9.2/3.6 ms, flip angle = 15°, matrix = 288 × 256, Field of view (FOV) = 240 × 240 mm, slice thickness = 1.6 mm, number of slices = 196, and scan time = 6 min 33 s. Fluid-attenuated inversion recovery (FLAIR) images were obtained with a fast spin-echo sequence with the following parameters: TR/TE = 8000/128 ms, matrix = 288 × 192, FOV = 240 × 240 mm, slice thickness = 4.0 mm, number of slices = 39, and scan time = 4 min 26 s.

**Fig. 1:**
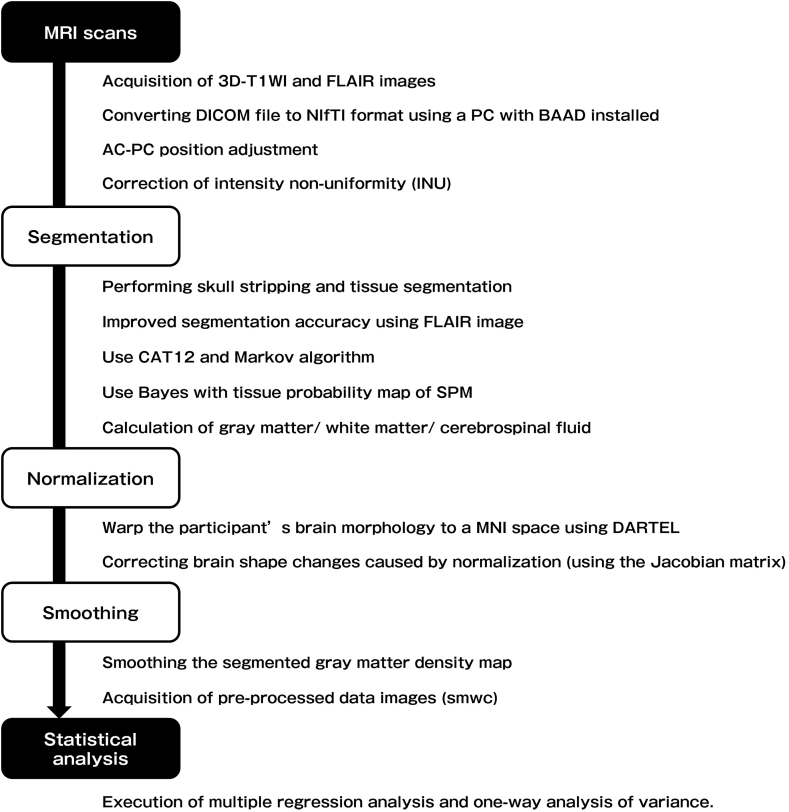
Overview of the VBM preprocessing procedure using BAAD in this study. Caption: VBM: Voxel-based morphometry, BAAD: Brain Anatomical Analysis using DARTEL, 3D-T1WI: 3D-T1-weighted images, FLAIR: fluid-attenuated inversion recovery, AC-PC: anterior commissure-posterior commissure, INU: intensity non-uniformity, CAT: computational anatomy toolbox, SPM: statistical parametric mapping, MNI: Montreal Neurological Institute. More information about VBM preprocessing using BAAD can be found elsewhere (Shiino et al., 2021; Syaifullah et al., 2021).

Data preprocessing obtained from BRAVO images was performed using the Brain Anatomical Analysis using DARTEL (BAAD) ver.4.4.0 (https://www.shiga-med.ac.jp/hqbioph/iBaad/page0.html). BAAD is a VBM-support software developed based on Statistical Parametric Mapping (SPM) 12 (Wellcome Trust Centre for Neuroimaging, UK). It includes various features such as automatic correction along the anterior commissure-posterior commissure axis, high-precision segmentation using maximum-likelihood estimation, maximum a-posteriori algorithms, and integration of FLAIR images. Additionally, BAAD enables the execution of SPM and related programs without requiring MATLAB (MathWorks, Natick, MA, USA). For this study, the 3D-T1WI and FLAIR images were processed with BAAD for segmentation, normalization, and smoothing. The voxel size was resampled to 1.5 × 1.5 × 1.5 mm, and total intracranial volume (TIV) was concurrently calculated. An overview of the preprocessing workflow using BAAD is illustrated in Fig. 1. Further details about BAAD can be found elsewhere (Shiino et al., 2021; Syaifullah et al., 2021).

### 2.4 Statistical analysis

The basic attributes of the subjects (age, sex, and educational level), TIV, and MMSE scores were calculated as frequencies or mean ± standard deviation to confirm their distribution. For intergroup comparisons among the three groups stratified by total MMSE score (NC, MCI, and D groups), one-way analysis of variance (ANOVA), Kruskal-Wallis test, and χ^2^ test were used. If significant differences were found, post-hoc tests (Bonferroni correction for multiple comparisons and residual analysis) were performed. Sex was treated as a nominal scale (0: female, 1: male), and education was treated as an ordinal scale (1: elementary/middle school graduate, 2: high school graduate, 3: vocational school/junior college graduate, 4: university/graduate school graduate) in the statistical analysis. The significance level α for statistical tests was set at 0.05, and IBM SPSS Statistics ver.25 (IBM Corp, Armonk, NY, USA) was used for the statistical analysis.

For neuroimaging analysis, we used SPM 12 within BAAD to conduct two types of whole-brain statistical analyses (absolute threshold masking = 0.1). First, a multiple regression analysis was performed with MMSE total score as the dependent variable and local brain volume (from the whole-brain analysis) as the independent variable, adjusting for age, sex, education, and TIV as covariates. Second, a one-way ANOVA was conducted to compare brain volume differences among the three groups based on MMSE scores (NC vs. MCI groups, and MCI vs. D groups), with age, sex, education, and TIV as covariates. For these analyses, multiple comparisons were corrected using Family-Wise Error (FWE) correction, with a significance level set at p < 0.05. The minimum cluster size was set at 40 contiguous voxels. As a sensitivity analysis, cluster-level correction was also performed using Threshold-Free Cluster Enhancement (TFCE), which considers both signal intensity and cluster extent for each voxel (5000 permutations were conducted) (Supplementary materials). Significant regions identified in the comparisons between the NC and MCI groups, as well as between the MCI and D groups, were extracted as regions of interest using MarsBaR (https://marsbar-toolbox.github.io/) in SPM 12 and overlaid on MRIcroGL templates (https://www.nitrc.org/projects/mricrogl).

## 3 Results

The study included 510 participants (286 women and 224 men) with a mean age ± standard deviation of 78.17 ± 9.09 years (women: 79.18 ± 8.95 years, men: 76.89 ± 9.12 years). Participants’ educational levels were as follows: 149 (29.2 %) completed elementary or junior high school, 288 (56.5 %) completed high school, 26 (5.1 %) completed vocational school or junior college, and 47 (9.2 %) completed university or graduate school. Intergroup comparisons among the NC, MCI, and D groups revealed significant differences in age (F_2,507_ = 32.77, p < 0.001) and educational level (H_2_ = 40.79, p < 0.001), but no significant difference was found in sex distribution (χ^2^_2_ = 2.82, p = 0.24, n.s.). Pairwise comparisons indicated that the mean age of the NC group was significantly lower than that of both the MCI and D groups (p < 0.05), and the MCI group had a significantly lower mean age than the D group (p < 0.05). The NC and MCI groups also had a significantly higher educational level compared to the D group (p < 0.05).

The basic attributes of the participants, TIV, total MMSE scores, and sub-item scores are presented in Table 1. In the comparisons between the three groups, significant effects were observed for TIV ((F_2,507_ = 3.82, p < 0.05), with the NC group showing significantly higher TIV than the D group (p < 0.05). Significant differences were also found in total MMSE scores (H_2_ = 450.73, p < 0.001), with post-hoc comparisons indicating that the NC group had significantly higher MMSE scores than the MCI and D groups (p < 0.05), and the MCI group had significantly higher scores than the D group (p < 0.05), confirming a stepwise decline in cognitive function from NC to MCI to D groups.

**Table 1:** Basic characteristics of the participants and MMSE results.

	Total: 510	NC: 199	MCI: 189	D: 122	statistics
Age	78.17 ± 9.09	74.59 ± 9.73	79.33 ± 7.43	82.22 ± 8.17	F_2,507_ = 32.77 ∗/∗∗/∗∗∗
Sex (female/male)	286/224	105/94	105/84	76/46	χ^2^_2_ = 2.82 n.s.
Educational level (1–4)	1.94 ± 0.84	2.20 ± 0.86	1.85 ± 0.80	1.66 ± 0.77	H_2_ = 40.79 ∗∗/∗∗∗
TIV	1479.25 ± 136.88	1498.58 ± 140.26	1473.29 ± 130.22	1456.95 ± 138.21	F_2,507_ = 3.82 ∗∗∗
MMSE total (/30)	25.41 ± 4.42	29.03 ± 0.78	25.68 ± 1.10	19.10 ± 4.17	H_2_ = 450.73 ∗/∗∗/∗∗∗
Orientation to place	4.22 ± 1.27	4.89 ± 0.34	4.50 ± 0.74	2.69 ± 1.58	H_2_ = 180.39 ∗/∗∗/∗∗∗
Orientation to time	4.61 ± 0.82	4.99 ± 0.07	4.72 ± 0.51	3.81 ± 1.22	H_2_ = 216.41 ∗/∗∗/∗∗∗
Registration	2.88 ± 0.41	2.98 ± 0.14	2.93 ± 0.33	2.64 ± 0.64	H_2_ = 56.64 ∗∗/∗∗∗
Attention/calculation	3.48 ± 1.52	4.67 ± 0.60	3.23 ± 1.20	1.92 ± 1.44	H_2_ = 255.80 ∗/∗∗/∗∗∗
Recall	2.19 ± 1.00	2.80 ± 0.46	2.20 ± 0.84	1.19 ± 1.06	H_2_ = 191.89 ∗/∗∗/∗∗∗
Naming	1.99 ± 0.15	2.00 ± 0.00	1.99 ± 0.10	1.96 ± 0.27	H_2_ = 4.74 n.s.
Repetition	0: 10.2 %	0: 2.5 % (−)	0: 9.5 %	0: 23.8 % (+)	χ^2^_2_ = 37.48
	1: 89.8 %	1: 97.5 % (+)	1: 90.5 %	1: 76.2 % (−)	
3 step commands	2.39 ± 0.75	2.75 ± 0.46	2.35 ± 0.70∗	1.86 ± 0.88∗∗	H_2_ = 102.07 ∗/∗∗/∗∗∗
Reading	0: 3.5 %	0: 0.5 % (−)	0: 0.5 % (−)	0: 13.1 % (+)	χ^2^_2_ = 43.27
	1: 96.5 %	1: 99.5 % (+)	1: 99.5 % (+)	1: 86.9 % (−)	
Writing	0: 8.6 %	0: 1.0 % (−)	0: 5.3 % (−)	0: 26.2 % (+)	χ^2^_2_ = 65.29
	1: 91.4 %	1: 99.0 % (+)	1: 94.7 % (+)	1: 73.8 % (−)	
Construction	0: 10.6 %	0: 2.0 % (−)	0: 10.1 %	0: 25.4 % (+)	χ^2^_2_ = 43.83
	1: 89.4 %	1: 98.0 % (+)	1: 89.9 %	1: 74.6 % (−)	

MMSE: mini-mental state examination, TIV: total intracranial volume. Age, educational level (1: elementary or middle school graduate, 2: high school graduate, 3: junior college or vocational school graduate, 4: university or graduate school graduate), MMSE total score and seven subtest scores (Orientation to place/time, Registration, Attention/calculation, Recall, Naming, and 3 step command) were presented as mean ± standard deviation, while sex and MMSE four subtest scores (Repetition, Reading, Writing, and Copying) was expressed as categorical data and percentage data (0: no points/1: points awarded). ∗: Significant difference between NC and MCI groups (p < 0.05), ∗∗: Significant difference between MCI and D groups (p < 0.05) respectively, ∗∗∗: Significant difference between NC and D groups (p < 0.05) respectively. (+): Cells with adjusted standardized residual values greater than or equal to 1.96 (p < 0.05), (−): Cells with adjusted standardized residual values greater than or equal to −1.96 (p < 0.05). All tests except for sex and the Naming on MMSE score were significant.

In the analysis of MMSE sub-item scores, significant differences were observed in the following areas: Orientation to place (H_2_ = 180.39, p < 0.001), Orientation to time (H_2_ = 216.41, p < 0.001), Registration (H_2_ = 56.64, p < 0.001), Attention/Calculation (H_2_ = 255.80, p < 0.001), Recall (H_2_ = 191.89, p < 0.001), Repetition (χ^2^_2_ = 37.48, p < 0.001), 3-step commands (H_2_ = 102.07, p < 0.001), Reading (χ^2^_2_ = 43.27, p < 0.001), Writing (χ^2^_2_ = 65.29, p < 0.001), and Construction (χ^2^_2_ = 43.83, p < 0.001). No significant difference was found in Naming (H_2_ = 4.74, p = 0.09, n.s.). Detailed results of multiple comparisons and residual analysis of subitems are shown in Table 1.

**Fig. 2:**
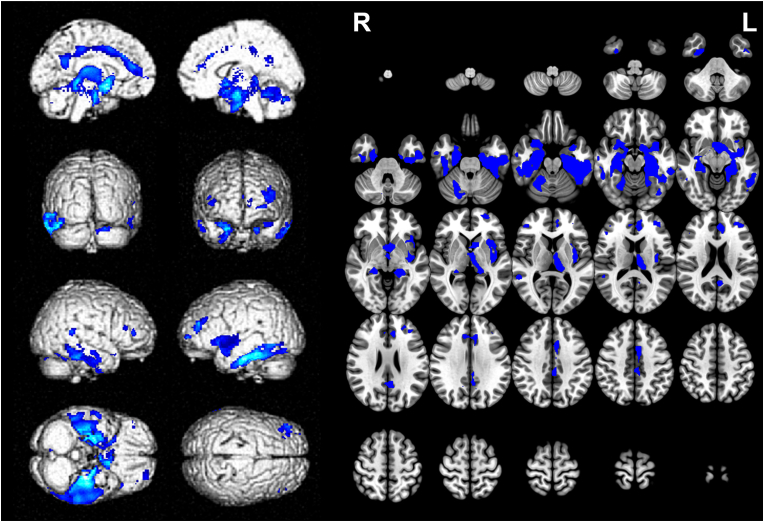
Localized brain regions indicating a positive correlation with MMSE total score. Caption: FWE-corrected p < 0.05 (k = 40), with age, gender, educational level, and total intracranial volume as covariates. L = left, R = right.

VBM analysis of structural MRI revealed specific brain regions that were positively correlated with MMSE total score. These regions included clusters extending from limbic system areas such as the left amygdala, left hippocampus, and left parahippocampal gyrus, to the left inferior temporal gyrus, clusters spreading from the left anterior cingulate cortex (ACC) to the left precuneus, bilateral middle/inferior frontal gyri, and the right temporal gyrus (FWE-corrected p < 0.05, k = 40) (Fig. 2 & Table 2). Notably, the left amygdala (MNI coordinates [x, y, z]: [−20, 5, −17], t = 9.33), left parahippocampal gyrus/hippocampus (MNI coordinates [x, y, z]: [−26, −26, −20], t = 10.36), and left inferior temporal gyrus (fusiform gyrus) (MNI coordinates [x, y, z]: [−39, −26, −21]) showed the highest correlations with the decline in MMSE scores. Sensitivity analysis using TFCE confirmed significant associations in all regions except the right middle frontal gyrus and right temporal gyrus (see supplementary material: Table 1).

**Table 2:** Localized brain regions indicating a positive correlation with MMSE total score.

Cluster index	Number of voxels	MNI coordinates (x, y, z)	t-score	Localized brain regions
Cluster 1	25,530∗	−26, −26, −20	10.36∗	L parahippocampal gyrus/hipocampus
		−39, −26, −21	9.46∗	L inferior temporal gyrus (fusiform gyrus)
		−20, 5, −17	9.33∗	L amygdala
Cluster 2	2820∗	−8, 38, 18	6.59∗	L anterior cingulate gyrus
		−8, 8, 42	6.37∗	L middle cingulate gyrus
		−5, −54, 23	5.75∗	L precuneus
Cluster 3	120∗	−26, 60, 2	5.87∗	L superior frontal gyrus
Cluster 4	622∗	−41, 39, 21	5.64∗	L middle frontal gyrus
		−33, 47, 15	5.49∗	L middle frontal gyrus
		−38, 33, 29	5.18∗	L middle frontal gyrus
Cluster 5	193∗	57, −45, 9	5.15∗	R middle temporal gyrus
Cluster 6	123∗	45, 42, 8	4.96∗	R middle frontal gyrus
Cluster 7	58∗	48, 33, 15	4.96∗	R inferior frontal gyrus (triangular part)
Cluster 8	231∗	60, −15, −18	4.90∗	R middle temporal gyrus
		57, −8, −26	4.89∗	R inferior temporal gyrus

MNI:Montreal Neurological Institute. ∗: Significant at FWE-corrected p < 0.05 (k = 40). L = left, R = right.

**Fig. 3:**
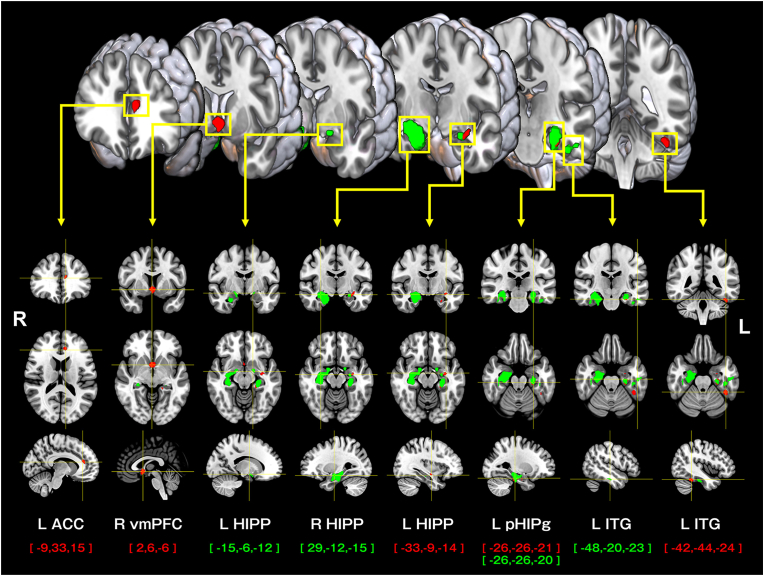
Changes in gray matter volume from Normal Cognition group to Dementia group. Caption: Red areas in the figure indicate regions where gray matter volume is reduced in the MCI group (MMSE 27–24) compared to the NC group (MMSE 30–28). Green areas show regions where gray matter volume is reduced in the D group (MMSE ≤23) compared to the MCI group (MMSE 27–24). The numbers in the curly brackets in the figure represent Montreal Neurological Institute (MNI) coordinates. NC: Normal cognition, MCI: Mild cognitive impairment, D: Dementia. L = left, R = right, ACC: anterior cingulate cortex, vmPFC: ventromedial prefrontal cortex, HIPP: hippocampus, pHIPg: parahippocampal gyrus, ITG: inferior temporal gyrus.

We conducted intergroup comparisons of decrease in GMV between two combinations of the three groups stratified by total MMSE scores: NC vs. MCI groups and MCI vs. D groups (Fig. 3 & Table 3). Intergroup comparisons between the NC and MCI groups, as well as between the MCI and D groups, showed significant GMV reductions in the MCI and D groups. In the NC vs. MCI comparison, significant reductions in GMV were found in the right ventromedial prefrontal cortex (vmPFC), left hippocampus, left parahippocampal gyrus, left inferior temporal gyrus (fusiform gyrus), and left ACC (FWE-corrected p < 0.05, k = 40). Sensitivity analysis using TFCE confirmed these findings and also indicated notable reductions in the right lingual gyrus and left cerebellum (see supplementary material: Table 2). In the MCI vs. D groups comparison, significant reductions in GMV were found in the bilateral hippocampus, parahippocampal gyrus, and left inferior temporal gyrus (fusiform gyrus) (FWE-corrected p < 0.05, k = 40). Sensitivity analysis using TFCE revealed significant reductions in GMV in the right hippocampus, parahippocampal gyrus, and right caudate nucleus, with significant differences primarily in the right hemisphere.

**Table 3:** The changes in brain volume from the Normal Cognition (NC) to Dementia (D) groups.

Cluster index	Number of voxels	MNI coordinates (x, y, z)	T-value	Localized brain regions
**NC > MCI**				
Cluster 1	254∗	2, 6, −6	5.59∗	R ventromedial prefrontal cortex
Cluster 2	136∗	−42, −44, −24	5.31∗	L inferior temporal gyrus (fusiform gyrus)
Cluster 3	275∗	−26, −26, −21	5.31∗	L parahippocampal gyrus
		−20, −36, −9	4.82∗	L parahippocampal gyrus
Cluster 4	105∗	−9, 33, 15	5.30∗	L anterior cingulate gyrus
Cluster 5	72∗	−33, −9, −14	5.02∗	L hippocampus
		−24, −8, −27	4.80∗	L hippocampus
**MCI > D**				
Cluster 1	2182∗	29, −12, −15	6.14∗	R hippocampus
		27, −21, −21	5.88∗	R parahippocampal gyrus
		14, −5, −14	5.65∗	R hippocampus
Cluster 2	671∗	−26, −26, −20	5.54∗	L parahippocampal gyrus
		−26, −14, −17	4.73∗	L hippocampus
Cluster 3	235∗	−48, −20, −23	5.27∗	L inferior temporal gyrus (fusiform gyrus)
		−39, −27, −26	4.82∗	L inferior temporal gyrus (fusiform gyrus)
Cluster 4	79∗	−15, −6, −12	4.98∗	L hippocampus

MNI:Montreal Neurological Institute. NC: Normal Cognition group (MMSE 30–28 points), MCI: Mild Cognitive Impairment group (MMSE 27–24 points), D: Dementia group (MMSE ≤23 points). T-value: test statistic, ∗: Significant at FWE-corrected p < 0.05 (k = 40). L = left, R = right.

No localized brain regions showed a significant negative correlation with total MMSE scores. Furthermore, no significant GMV decreases were found when comparing the NC group to the MCI group or the MCI group to the D group in the intergroup comparisons.

## 4 Discussion

### 4.1 Localized brain regions correlated with total MMSE scores

This study examined the decline in GMV positively associated with MMSE scores in a Japanese population. The results demonstrated that regions exhibiting GMV atrophy correlated with total MMSE scores—an index of overall cognitive function—were primarily located in the limbic system, including the hippocampus, amygdala, and parahippocampal gyrus, all of which play critical roles in memory function. Additionally, extensive cortical regions showed GMV reductions, including the medial and lateral prefrontal cortex (e.g., the cingulate gyrus and middle frontal gyrus), which are involved in cognitive control (MacDonald et al., 2000), as well as posterior medial (precuneus) and lateral (temporal gyrus) regions, which contribute to visuospatial imagery, episodic memory retrieval, self-referential processing, and consciousness (Jitsuishi and Yamaguchi, 2023). A prior study conducted in a French cohort reported similar findings regarding brain regions associated with total MMSE scores (Dinomais et al., 2016), supporting the cross-validity of our results. These regions are integral to the Papez circuit, a neural pathway primarily associated with memory function (Forno et al., 2021). Moreover, the medial temporal lobe, including the hippocampus and precuneus, is a key component of the Default Mode Network (DMN), which plays a crucial role in self-referential episodic memory formation (Greicius et al., 2004). Given that memory function decline is a hallmark of MCI and AD, lower MMSE scores are likely to reflect these impairments. However, previous research has also implicated additional brain regions, such as the bilateral caudate nucleus, bilateral insula, and the left superior parietal lobule extending to the angular gyrus, in cognitive impairment (Dinomais et al., 2016). These discrepancies may stem from several factors, including differences in sample size, participant characteristics (e.g., age, gender distribution, racial background), social environment, and imaging methodologies. Further studies incorporating larger, more diverse populations and standardized imaging protocols are warranted to elucidate these regional differences more comprehensively.

### 4.2 Regions of decreased GMV associated with cognitive decline

This study examined brain regions exhibiting GMV reductions that characterize different stages of cognitive decline from NC to MCI and further to D groups, utilizing VBM analysis of structural MRI. Group-wise comparisons were performed between the NC and MCI groups and between the MCI and D groups, stratified based on MMSE total score cutoffs. The results revealed significant GMV atrophy in the left hippocampus, parahippocampal gyrus, ACC, and vmPFC when comparing the NC and MCI groups. Further cognitive decline, as reflected in the comparison between the MCI and D groups, was associated with bilateral hippocampal atrophy and significant GMV reductions in the left inferior temporal gyrus, including the fusiform gyrus. These findings are consistent with previous research indicating that GMV loss in MCI and AD follows a region-specific pattern, with early degeneration primarily observed in the medial temporal lobe in MCI, followed by more widespread cortical involvement in AD (Derflinger et al., 2011; Minkova et al., 2017). The results of this study further support this pattern, as the observed GMV reductions in the medial temporal structures, including the hippocampus and parahippocampal gyrus, were particularly pronounced in the NC and MCI groups. In contrast, more extensive cortical atrophy, particularly in the inferior temporal gyrus and other neocortical regions, was more prominent in the MCI and D groups. This pattern of region-specific atrophy across different diagnostic groups aligns with the known trajectory of neurodegeneration in AD, reinforcing the hypothesis that MCI represents an intermediate stage in this continuum.

Previous studies have consistently reported atrophy in the hippocampus, parahippocampal gyrus, ACC, and inferior temporal gyrus in individuals with MCI and AD (Dinomais et al., 2016). Notably, our findings suggest that the right vmPFC emerges as a newly implicated region in the transition from the preclinical or NC stage to MCI. The vmPFC plays a crucial role in reward and value processing, decision-making, emotional regulation, and social cognition and serves as a key hub of the DMN, which is involved in self-referential processing (Greicius et al., 2004; Lopez-Persem et al., 2019). Damage to the right vmPFC has been associated with confabulation, a phenomenon characterized by spontaneous false episodic memories reported with high confidence (Lopez-Persem et al., 2019; Nieuwenhuis and Takashima, 2011). Additionally, this region is thought to integrate information from other limbic structures and suppress irrelevant information based on this integrated representation (Nieuwenhuis and Takashima, 2011). Therefore, atrophic decrease in GMV in the right vmPFC might be considered a notable brain region for early detection of amnestic MCI and its preceding state.

### 4.3 Limitations and future directions

This study analyzed over 500 Japanese individuals, identifying brain regions correlated with total MMSE scores. Additionally, by comparing three groups stratified by MMSE cutoff scores, we delineated brain regions exhibiting progressive GMV reductions with cognitive decline. However, several limitations must be acknowledged.

First, participants were not classified based on formal diagnostic criteria, necessitating further investigation by a neurologist to more precisely distinguish clinical diagnoses such as MCI and AD. Future studies should incorporate established diagnostic frameworks to enhance clinical applicability.

Second, our cross-sectional analysis using VBM revealed an asymmetric pattern of GMV atrophy, beginning in the left hippocampus and extending to the right during progression from NC to D. This left-dominant atrophy might be due to younger MCI and AD patients showing more rapid and pronounced atrophy (Fiford et al., 2018; Tabatabaei-Jafari et al., 2015), though previous studies have not reached consensus (Derflinger et al., 2011; Minkova et al., 2017; Zhang et al., 2021). Future research should integrate other biomarkers and longitudinal analyses to better predict asymmetric atrophy patterns and brain localization accurately.

Third, while MRI-based assessments provide valuable insights into structural brain changes, their cost and accessibility remain significant barriers to large-scale screening applications in many countries. However, Japan has the highest number of MRI scanners per capita under its universal health insurance system, making MRI relatively accessible even in rural areas (Matusmoto et al., 2015; Nature research custom media, 2024). This suggests that MRI-based cognitive assessments may be more feasible in Japan than in other healthcare settings. Nevertheless, the financial burden associated with MRI remains a consideration, and future studies should evaluate its cost-effectiveness for dementia screening in routine clinical practice.

Fourth, despite the MMSE's widespread use, it has limitations in detecting very mild cognitive disturbances due to its ceiling effect and susceptibility to educational bias. The Montreal Cognitive Assessment (MoCA) has been shown to be more sensitive in detecting early cognitive impairments (Pint et al., 2019; Sawada et al., 2023). However, MMSE remains the preferred tool in many clinical and research settings due to its simplicity and extensive validation. Additionally, MMSE-based classification facilitated direct comparisons with previous studies (Dinomais et al., 2016), providing a standardized analytical framework. To improve the detection of subtle cognitive changes during the NC-to-MCI transition, future studies should integrate MoCA or other refined neuropsychological assessments alongside MMSE.

Finally, recently developed computerized cognitive screening tools, which assess cognitive performance based on accuracy and response time, may provide promising alternatives for detecting very mild cognitive disturbances (Satoh et al., 2024; Sawada et al., 2023; Tsoy et al., 2021). These digital tools offer advantages such as automated administration, scalability, and greater sensitivity to subtle cognitive decline compared to traditional paper-based tests. Given their accessibility and efficiency, computerized cognitive assessments could complement MRI-based structural analyses by providing real-time cognitive performance data that can be correlated with GMV reductions in key brain regions. Future research should explore the integration of computerized cognitive assessments with VBM analysis of structural MRI, enabling a more precise evaluation of brain regions associated with cognitive decline. Such advancements could significantly refine early detection strategies and facilitate personalized intervention approaches for individuals at risk of dementia. Moreover, combining MRI, MoCA, and digital cognitive testing could establish a multimodal framework that enhances diagnostic accuracy while ensuring practical feasibility in diverse clinical settings.

### 4.4 Conclusion

Using VBM analysis of structural MRI, we identified brain regions positively correlated with total MMSE scores, including the bilateral frontal lobes, left medial frontal lobe, left hippocampal region, left ACC, and bilateral inferior temporal gyri. When comparing the three groups stratified by MMSE cutoff values (NC, MCI, and D), significant GMV reductions were in the left hippocampus, parahippocampal gyrus, left ACC, and right vmPFC in the MCI group compared to the NC group. Further GMV reductions were detected in the bilateral hippocampal regions and the left inferior temporal gyrus in the D group compared to the MCI group. These findings support VBM-based structural MRI as a valuable tool for detecting early brain atrophy in mild cognitive impairment, aiding in early diagnosis, timely intervention, and improved clinical management. Future research should focus on expanding studies and integrating biomarkers to enhance diagnostic accuracy.

## CRediT authorship contribution statement

**Yoichi Sawada:** Writing – review & editing, Writing – original draft, Validation, Methodology, Funding acquisition, Formal analysis, Data curation, Conceptualization. **Toru Satoh:** Writing – review & editing, Writing – original draft, Visualization, Validation, Supervision, Project administration, Methodology, Investigation, Formal analysis, Data curation, Conceptualization. **Hideaki Saba:** Methodology, Investigation, Formal analysis, Data curation. **Yoshiki Kato:** Methodology, Investigation, Formal analysis, Data curation. **Tomoko Kuwada:** Methodology, Data curation. **Sayoko Shima:** Methodology, Data curation. **Kana Murakami:** Methodology, Data curation. **Megumi Sasaki:** Methodology, Data curation. **Yudai Abe:** Methodology, Data curation. **Kaori Harano:** Writing – review & editing, Methodology, Conceptualization.

## Declaration of competing interest

The authors have no conflicts of interest to declare.

## Data Availability

Data will be made available on request.
