# Supplementary material for: Assessment of localized brain regions correlated with MMSE using VBM analysis of structural MRI in a Japanese sample
Source: Neuroimage Rep. 2025 Apr 29;5(2):100264. doi: 10.1016/j.ynirp.2025.100264 (PMC12172859; doi:10.1016/j.ynirp.2025.100264)
Supplement: Multimedia component 1 [file mmc1.docx]

**Supplementary table 1. Sensitivity analysis: localized brain regions indicating a positive correlation with MMSE total score by TFCE.**

| Cluster index | Number of voxels | MNI coordinates  (x, y, z) | TFCE value | Localized brain regions |
| --- | --- | --- | --- | --- |
| Cluster 1 | 119773* | -26, -26, -20  -23, -32, -14  -27, -14, -17 | 19703.91  19446.43  18138.47 | L parahippocampal gyrus  L hippocampus  L inferior temporal gyrus (fusiform gyrus)  L parahippocampal gyrus  L hippocampus  L inferior temporal gyrus (fusiform gyrus)  L hippocampus  L amygdala |
| Cluster 2 | 1* | -36, -35, 60 | 744.73 | L postcentral gyrus  L superior parietal lobule |
| Cluster 3 | 6* | -33, -35, 59 | 744.28 | L postcentral gyrus  L superior parietal lobule |

MNI：Montreal Neurological Institute. This displays brain local regions significantly associated with the total score of MMSE. *: Significant at FWE_TFCE_-corrected p < 0.05 (the number of permutation tests was set at 5000). L = left, R = right.

**Supplementary table 2. Sensitivity analysis: the changes in brain volume from the Normal Cognition (NC) to Dementia (D) groups by TFCE.**

| Cluster index | Number of voxels | MNI coordinates  (x, y, z) | TFCE value | Localized brain regions |
| --- | --- | --- | --- | --- |
| **NC > MCI** |  |  |  |  |
| Cluster 1 | 41469* | -26, -26, -21  -20, -35, -11  -42, -44, -26 | 2642.80*  2579.96*  2512.49* | L parahippocampal gyrus/ hippocampus  L parahippocampal gyrus/ hippocampus  L inferior temporal gyrus (fusiform gyrus) |
| Cluster 2 | 1850* | -9, 35, 14  -12, 32, 27  3, 44, 17 | 1277.33*  1047.08*  964.66* | L anterior cingulate gyrus  L anterior cingulate gyrus  L anterior cingulate gyrus |
| Cluster 3 | 305* | -39, 36, 24 | 815.66* | L middle frontal gyrus |
| Cluster 4 | 16* | -50, 32, 0 | 743.77* | L inferior frontal gyrus tri |
| Cluster 5 | 1* | 20, -80, -6 | 736.13* | R lingual gyrus |
| Cluster 6 | 7* | -35, -42, -44 | 703.50* | L cerebellum |
| **MCI > D** |  |  |  |  |
| Cluster 1 | 47572* | 29, -12, -15  27, -21, -21  14, -5, -14 | 3815.56*  3778.09*  3491.79* | R hippocampus  R parahippocampal gyrus  R hippocampus |
| Cluster 2 | 1* | 11, 20, -5 | 728.76* | R caudate |

MNI：Montreal Neurological Institute. NC: Normal Cognition group (MMSE 30-28 points), MCI: Mild Cognitive Impairment group (MMSE 27-24 points), D: Dementia group (MMSE ≤ 23 points). TFCE value: test statistic, *: Significant at FWE_TFCE_-corrected p < 0.05 (the number of permutation tests was set at 5000). L = left, R = right.
